# Stauffer syndrome, clinical implications and knowledge gaps, does size matter? Case report

**DOI:** 10.1186/s12894-020-00671-w

**Published:** 2020-07-20

**Authors:** Julian Chavarriaga, Nayib Fakih, Juan Cataño, Catalina Villaquiran, Santiago Rodriguez, German Patino

**Affiliations:** Division of Urologic Oncology, Pontificia Universidad Javeriana, Hospital Universitario San Ignacio, Carrera 7 No. 40 – 62, Bogotá, Colombia 11011

**Keywords:** Carcinomas, renal cell, Paraneoplastic syndrome, Liver dysfunctions, Jaundice, Cholestatic, Case report

## Abstract

**Background:**

Stauffer Syndrome (SS) is a paraneoplastic disorder associated with renal cell carcinoma (RCC). First described by Herbert Maurice Stauffer in 1961, it is characterized by hepatic dysfunction in the absence of metastasis, and elevated alkaline phosphatase, aminotransferases and prolonged prothrombin time. Initial reports did not include jaundice as an inclusion criterion. We aim to report the rare SS jaundice variant in a patient with a small renal mass and review the literature to determine if the size of the renal mass could influence the development of the SS.

**Case presentation:**

The aim of this article is to present the case of a 53-year-old male with Stauffer syndrome jaundice variant secondary to a 2.5 cm renal mass, treated with laparoscopic radical nephrectomy with complete resolution of the syndrome.

**Conclusions:**

This syndrome is yet to be fully understood, and as far as the evidence shows, size does not matter. This entity should always be in mind when encountered with a patient with liver dysfunction and jaundice with a suspicion or confirmed diagnosis of a renal mass. To date, and to our knowledge, there have been eleven reported cases of paraneoplastic cholestatic jaundice syndrome including the current case. Six cases presented in patients with small renal mases (< 4 cm), curiously there were not a laterality tendency.

## Background

Renal cell carcinoma (RCC) accounts for 2% of all cancers. The classic diagnostic triad of palpable abdominal mass, hematuria and right flank pain is only present in 10% of patients [1]. It has been related to many paraneoplastic syndromes, ranging between neuromyopathy, polycythemia, malignant hypercalcemia, anemia, and liver dysfunction [2]. Stauffer syndrome (SS) is one of the most characteristic paraneoplastic syndromes associated with RCC. First described in 1961 by Herbert Stauffer, the five originally reported cases presented: hypernephroma (nowadays RCC), hypoalbuminemia, hypergammaglobulinemia, high alkaline phosphatase (ALP), prolonged prothrombin time (PT) with normalization of liver function tests (LFTs) after tumor resection [3]. The incidence in patients with RCC varies between 3 and 6% [4, 5]. Development of SS has been associated with bad prognosis [4, 5], a few case reports of SS include jaundice as a diagnostic variable [6]. Most case reports have usually shown very large renal masses [7].

The aim of this article is to present the rare case of a 53-year-old male with a small renal mass (SRM), who developed SS with jaundice and liver dysfunction, and underwent laparoscopic radical nephrectomy with complete resolution of the syndrome features after treatment of the primary lesion.

## Case presentation

A 53-year-old male presented to the emergency department (ER) with right upper quadrant abdominal pain, jaundice, pruritus, choluria and acholia. His complete blood count (CBC) was normal. Abnormal LFTs: total bilirubin of 19.91 mg/dl, Aspartate Transaminase (AST) 418 U/L, Alanine transaminase (ALT) 695 U/L. Lactate Dehydrogenase (LDH), albumin, prothrombin and thromboplastin times were normal, INR was 1. HIV, hepatitis B, C, Epstein-Barr virus (EBV) antibodies, and peripheral blood smear were within normal ranges. Autoimmune disorders comprehensive panel was normal. Past medical history was non remarkable and acetaminophen toxicity was ruled out at ER admission.

**Fig. 1:**
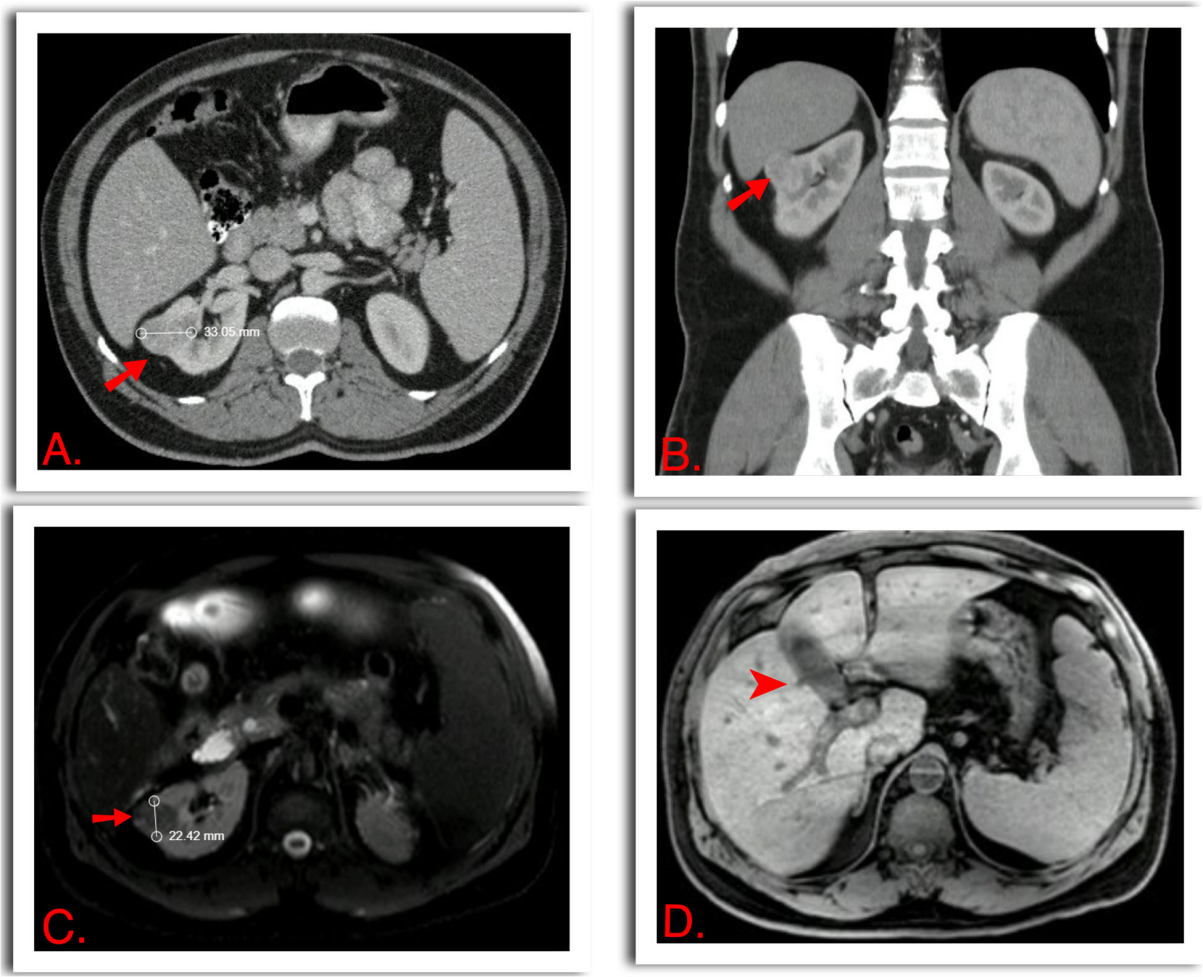
**a** and **b**. Computed tomography showed a solid exophytic mass (arrow) measuring 24 x29x33 mm in the right kidney, RENAL score 9x. **c.** Contrast enhanced MRI, T2W, showed a 22 mm renal mass in the long axis, which enhanced with IV contrast. **d**. Hepato-Splenomegaly characteristic of the Stauffer syndrome, Gallbladder and hepatic biliary ducts were not dilated (Arrow head)

Radiological work up, with computerized tomography (CT) showed a 24 × 29 × 33 mm renal mass, at the interpolar region of the right kidney with a RENAL nephrometry score of 9 (moderate complexity), with hepatosplenomegaly and without signs of choledocholithiasis, pancreatic masses or metastasis. (Figure 1) Chest CT was unremarkable. The magnetic resonance imaging (MRI) cholangiopancreatography and portal doppler ultrasound ruled out any other structural disease.

**Fig. 2:**
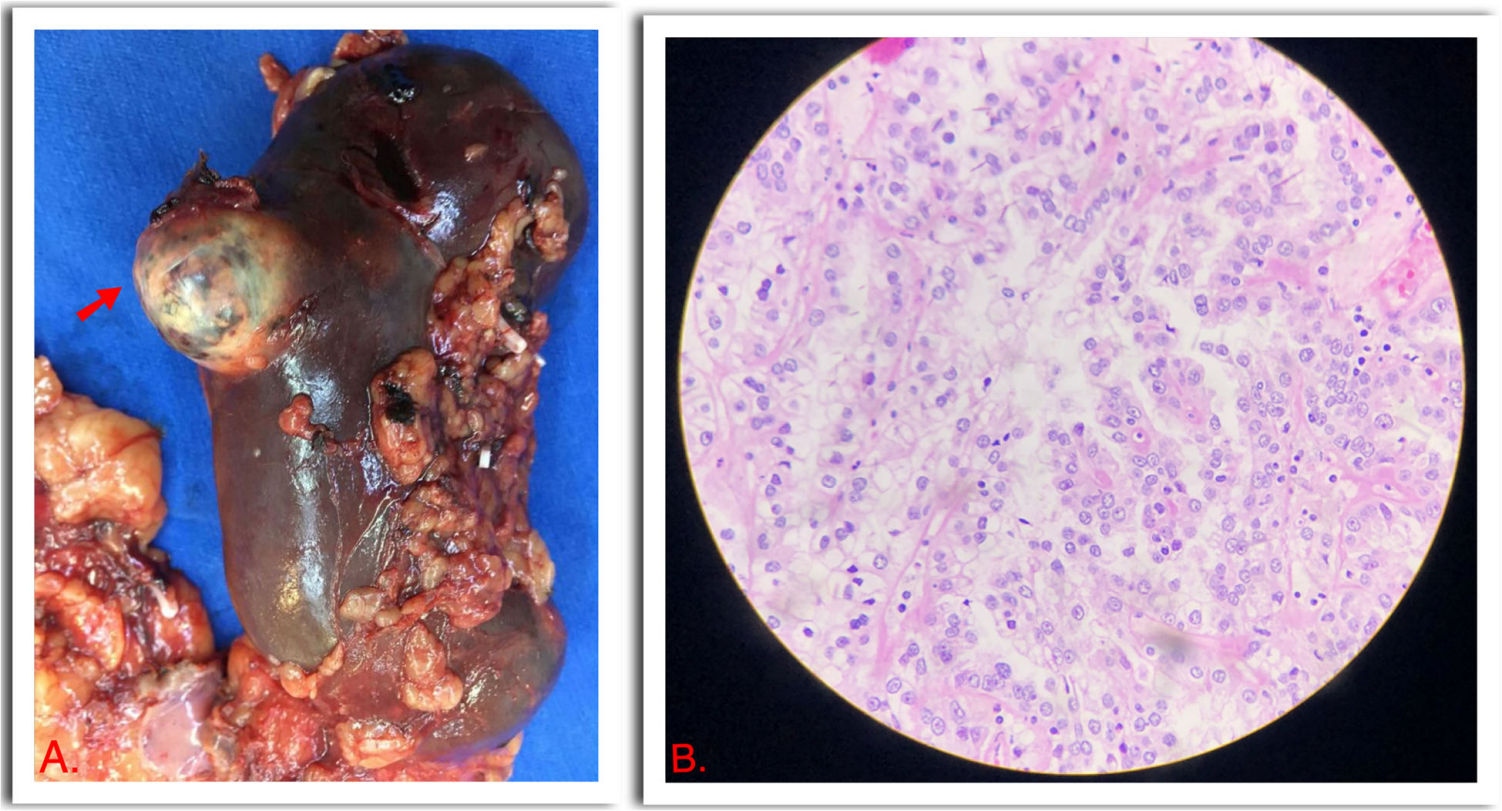
**a.** Macroscopic surgical pathology showing an exophytic solid renal mass measuring 2.5 cm. (Arrow) **b.** Hematoxylin-eosin, 40-100x magnification. Tumor composed of compact alveolar structures, cells with clear cytoplasm and prominent nucleoli, characteristic of the clear cell carcinoma variant histology

As the bilirubin levels kept increasing and after ruling out all differential diagnosis, Gastroenterology and Hepatology got consulted and they suggested a jaundice variant of the Stauffer paraneoplastic syndrome, patient was scheduled for urgent laparoscopic radical nephrectomy instead of a nephron-sparing approach considering that liver dysfunction could imply a higher surgical bleeding risk. Surgery was performed without complications, total bleeding was 100 ml. Postoperative course was uneventful, and in postoperative day 3, upon discharge, total bilirubin was 3.31 mg/dL, alkaline phosphatase 181 U/L, AST 93 U/L, ALT 231 U/L. Surgical pathology revealed a pT1a, 2.5 cm clear cell carcinoma, WHO/ISUP nucleolar grade 2. (Figure 2) At 6 months follow up improvement of LFTs has remained normalized and hepatosplenomegaly has resolved, confirmed with US.

## Discussion and conclusions

Stauffer syndrome is an uncommon paraneoplastic syndrome [4, 5]. It is usually associated with RCC, but it can present along with other types of malignancies such as soft-tissue sarcomas, prostate cancer and malignant lymphoproliferative diseases [3, 5]. Stauffer syndrome can be divided into a classic presentation and jaundice variant. The latter is characterized by high alkaline phosphatase, thrombocytosis, elevated erythrocyte sedimentation rate, hyperbilirubinemia, hepatosplenomegaly, in some cases, abnormal coagulation profile and hypoalbuminemia, jaundice, choluria, pruritus are clinical features of this syndrome [3, 5, 8]. Our case corresponds to the aforementioned jaundice variant.

Interleukin-6 (IL-6) has been proposed to play a major role in the pathophysiology of this syndrome [3, 5, 9]. Bhangoo, et al suggested that cholestasis could be secondary to pro-inflammatory activity of IL-6 cytokine which causes elevation of C-reactive protein, haptoglobin and inhibits the hepatobiliary transporter gene expression which impairs biliary outflow [9, 10]. Patients with detectable serum IL-6 had significantly higher hepatic compromise with elevated LFTs [9]. Karakolios et al. conducted a study treating patients with SS with anti-IL-6 monoclonal antibodies and found that it reversed most of the biochemical abnormalities of these patients [11]. Despite these findings, the precise mechanism of the disease remains poorly understood, the association of this condition with IL-6 remains speculative and other humoral mediators have also been proposed such as an increase in lysosomal enzyme activity in liver cells of patients with RCC, which has effects on the liver and hematopoietic system [5] and the lack of humoral secretion inhibition by suramin [12].

Autopsy findings have revealed hepatic sinusoidal dilation without congestion of the central hepatic vein or hepatocellular necrosis [3]. Interestingly in more recent years, immune checkpoint inhibitors such as Programmed death ligand 1/Programmed cell death protein1 (PD-L1/PD-1) have been approved as treatment for metastatic RCC. These medications, with immune-mediated mechanisms of action, may explain their efficacy and correlation to the pathophysiology of Stauffer Syndrome [3, 5, 13]. It has been reported that IL-6/JAK1 pathway promotes cancer immune evasion through phosphorylation of PD-L1 (Tyr112) [13].

A rare variant of the syndrome presents initially with jaundice, as in this case. It was first reported by Dourakis, et al in 1997 [8]. Two patients presented to the emergency department complaining of jaundice, and one of them had also right upper quadrant pain. Both patients had right renal masses that once were removed, symptoms improved quite rapidly. In 2005 Giannakos, et al also reported a 73-year-old male with jaundice, pruritus, choluria and hepatomegaly. A 3 cm right lower pole renal mass was seen in a CT scan, and once removed, symptoms resolved as well.

To date, and to our knowledge, there have been eleven reported cases of paraneoplastic cholestatic jaundice syndrome in the literature including the current case [5–8]. Clinical characteristics and laboratory and imaging work up is shown on Table 1 [5]. Six cases presented in patients with SRM (< 4 cm), curiously there were not a laterality tendency, 6 RCCs were located in the right kidney and 5 in the left and most (8) were clear cell histology [5–8].

**Table 1:** Clinical characteristics of patients with Stauffer syndrome variant with cholestatic jaundice due to RCC

Clinical studies	n	Age (Years)	Laterality	Tumor size (cm)	AST	ALT	ALP	INR	Bilirrubin	Treatment	Histology
**Chavarriaga et al. (2020)**	***n*** **= 1**	**53**	**Right**	**3**	**418**	**695**	**337**	**1**	**19.91**	**RLN**	**RCCcc**
**Dewana et al. (2019)** [7]	***n*** **= 1**	**32**	**Right**	**19**	**74**	**30**	**253**	**3.2**	**9.25**	**ORN**	**SCS**
**Gremida et al. (2017)** [14]	***n*** **= 1**	**55**	**Left**	**2.5**	**29**	**34**	**327**	**1.6**	**20.9**	**RLN**	**RCCcc**
**Puga et al. (2015)** [15]	***n*** **= 1**	**69**	**Right**	**6.2**	**57**	**50**	**367**	**1.2**	**22.9**	**RLNU**	**pRCC**
**Fernandez et al. (2012)** [16]	***n*** **= 1**	**70**	**Left**	**NR**	**672**	**397**	**222**	**2.04**	**10.3**	**RLN**	**NR**
**Tomadini et al. (2010)** [17]	***n*** **= 1**	**51**	**Left**	**4**	**N**	**N**	**7860**	**N**	**13.6**	**ORN**	**RCCcc**
**Mazokopakis et al. (2007)** [18]	***n*** **= 1**	**50**	**Left**	**> 7**	**227**	**260**	**187**	**2.9**	**2.1**	**ORN**	**RCC**
**Morla et al. (2006)** [6]	***n*** **= 1**	**39**	**Left**	**4**	**146**	**24**	**219**	**1.12**	**16.8**	**ORN**	**RCCcc**
**Giannakos et al. (2005)** [19]	***n*** **= 1**	**73**	**Right**	**3**	**240**	**123**	**287**	**NR**	**38.1**	**ORN**	**RCCcc**
**Dourakis et al. (1997)** [8]	***n*** **= 1**	**65**	**Right**	**2.5**	**158**	**426**	**163**	**NR**	**6.2**	**ORN**	**RCCcc**
**Dourakis et al. (1997)** [8]	***n*** **= 1**	**48**	**Right**	**> 10**	**132**	**334**	**193**	**NR**	**2.8**	**ORN**	**RCCcc**

*RCC* Renal cell carcinoma, *RCCcc* Clear Cell, *pRCC* Papillary, *SCS* Synovial Cell Sarcoma, *ALP* Alkaline phospatase, *ALT* alanine aminotransferase, *AST* aspartate transaminase, *RLN* Radical laparoscopic Npehrectomy, *ORN* Open Radical Nephrectomy, *NR* Not reported, *INR* international normalized ratio of prothrombin time of blood coagulation, *N* Normal

This syndrome is yet to be fully understood, and as far as the evidence shows, size does not matter. This entity should always be in mind when encountered with a patient with liver dysfunction and jaundice with a suspicion or confirmed diagnosis of a renal mass. Efforts should be made to clarify the precise pathophysiology of this syndrome, molecular studies in patients suffering from SS and serum tests looking for pro-inflammatory cytokines should be taken into account when diagnosing and treating this rare syndrome, all these to finally define targeted therapy while the patient awaits the surgical treatment given the considerable risk of bleeding in a patient with liver dysfunction.

## Data Availability

The datasets used and/or analysed during the current study are available from the corresponding author on reasonable request.

## Abbreviations

SS: Stauffer Syndrome
RCC: Renal cell carcinoma
ALP: Alkaline phosphatase
PT: Prothrombin time
LFTs: Liver function tests
SRM: Small renal mass
CBC: Complete blood count
ALT: Alanine transaminase
AST: Aspartate Transaminase
LDH: Lactate Dehydrogenase
CT: Computerized tomography
MRI: Magnetic resonance imaging
IL-6: Interleukin-6
PD-L1: Programmed death-ligand 1
PD-1: Programmed cell death protein 1
